# National action plan on antimicrobial resistance in selected Asia-Pacific low- and middle-income countries: Perspectives of One Health stakeholders

**DOI:** 10.1016/j.onehlt.2025.101259

**Published:** 2025-10-30

**Authors:** Yasodhara D. Gunasekara, Kirsten E. Bailey, Ri O. Scarborough, Anna E. Sri, Mauricio J.C. Coppo, James R. Gilkerson, Glenn F. Browning, Laura Y. Hardefeldt

**Affiliations:** a Asia-Pacific Centre for Animal Health, Melbourne Veterinary School, The University of Melbourne, Parkville, Australia; b National Centre for Antimicrobial Stewardship, The University of Melbourne, Parkville, Australia; c Instituto One Health, Facultad de Ciencias de la Vida, Universidad Andres Bello, Concepcion, Biobio, Chile

**Keywords:** Antimicrobial stewardship, Antimicrobial use surveillance, Antimicrobial resistance surveillance, Implementation

## Abstract

Antimicrobial resistance (AMR) is a One Health problem worldwide, with low and middle-income countries (LMICs) identified as hotspots for AMR development. Countries have created National Action Plans on AMR (NAP-AMR) to address the growing problem; however, studies on the progression of NAP-AMR implementation are scarce. This study explores the current status of the implementation and impact of NAP-AMR in selected LMICs in the Asia-Pacific region through a One Health lens. In total, 102 experts from Bhutan, Nepal, Pakistan, Timor-Leste and Papua New Guinea were enrolled as the study population. Their perspectives on the level of implementation of NAP-AMR were collected via an online questionnaire, and an 80 % response rate was obtained. Participants felt that NAP-AMR implementation was more advanced in human health than in animal or environmental health. Stakeholders perceived to be well informed also tended to be engaged more actively in implementing the responsibilities outlined in the NAP-AMR. Nearly all participants believed that their country needed local antimicrobial prescribing guidelines for humans (94 %) and animals (86 %). Unrestricted access to antimicrobials and poor implementation status of surveillance systems across human, animal and environmental sectors were identified as areas where more progress is needed. Participants' beliefs about the current level of training and awareness related to AMR, as well as current AMR and AMU surveillance, was different from the Quadripartite AMR country self-assessment survey. Further studies need to be conducted to explore the barriers to NAP-AMR implementation in this setting. This study highlights the importance of systematic and transparent monitoring and evaluation frameworks across multiple One Health sectors, to assess progress on implementation of action plans and identify gaps for further investment or intervention. The importance of co-designing monitoring and evaluation frameworks with input from diverse AMR stakeholders across One Health domains was also identified as critical to ensuring the relevance of these frameworks and an equitable approach.

## Highlights

• Gaps exist in NAP-AMR implementation, especially in the environmental sector
• Substantial disagreement between the findings of this survey and the Quadripartite AMR country self-assessment survey
• Need for transparent monitoring and evaluation frameworks across the One Health

## 1 Background

Antimicrobial resistance (AMR) is one of the top ten global health problems and a barrier to achieving the United Nations (UN) sustainable development goals [[1], [2], [3]]. The AMR burden in low- and middle-income countries (LMICs) is disproportionately high compared to high-income countries (HICs), and LMICs may face exacerbated poverty due to the growing burden of AMR [4]. AMR is a One Health issue as the burden impacts humans, animals, and the environment [[5], [6], [7]].

To tackle the problem, the World Health Organisation (WHO) announced the Global Action Plan (GAP) on AMR in 2015 [8]. Later, the GAP-AMR was further endorsed by the Food and Agriculture Organisation (FAO), the World Organisation for Animal Health (WOAH) and the UN Environment Programme (UNEP) [9]. In 2017, 194 WHO member countries agreed to develop and implement a National Action Plan (NAP) on AMR [10]. By 2024, more than 90 % of these countries had developed a NAP-AMR, with some countries publishing a second revision [11]. The objectives of most NAP-AMRs align closely with the strategic objectives of the WHO GAP-AMR [12].

The vast majority of countries in the Asia-Pacific region have published NAP-AMR [13]. However, studies on their progression and level of implementation are not widely available [14]. Inappropriate use of antibiotics and poor awareness among prescribers and users were reported in this region [[15], [16], [17], [18]]. Also, projections indicate that by 2050, South Asia will report the highest death rate attributable to AMR, estimated at 114 deaths per 100,000 population [19,20].

The objective of this study was to explore the current status of the implementation and impact of NAP-AMR in LMICs in the Asia-Pacific region. Understanding the implementation, or barriers to implementation, of NAP-AMR in the Asia-Pacific region may help in curbing the impact of particularly in LMICs, where AMR is likely to have the most significant impact.

## 2 Methods

One group of experts who are uniquely positioned to assess perspectives on the current status of AMR awareness, training, and surveillance in their countries are Fleming Fund Fellows. The Fleming Fund is a UK aid program aimed at supporting LMICs to address AMR through a One Health approach. It supports countries in building sustainable surveillance systems, enhancing laboratory capacity, and training professionals to improve the detection and management of AMR, for example, through its Fleming Fund Fellowship Scheme. This scheme supports the professional development of technical experts to strengthen AMR or antimicrobial consumption and use surveillance and policy in LMICs across One Health domains. Fellows who worked in national institutions such as national reference laboratories, hospitals, and government agencies in LMICs were chosen through a competitive process. The Fellowships were delivered through a model of mentorship and on-the-job training, collaborative projects and intensive workshops, to support implementation of NAP-AMR, using a One Health approach. Convenience sampling was used to select the study population. Since Fleming fellows play a significant role in tackling AMR in their countries, their perspectives on the level of implementation of NAP-AMR in their countries were anticipated to be valuable. Participants in the Fleming Fund Fellowship program from Bhutan, Nepal, Pakistan, Timor-Leste and Papua New Guinea were invited by email to complete the survey. A questionnaire was developed by multiple members of the research team (YDG, KEB, ROS, MJCC, and LYH). The questionnaire [included in supplementary materials] consisted of four sections with predominantly closed-ended questions and few open-ended questions. The initial section asked demographic questions. The second section explored the respondents' perspectives on the format of, awareness of, engagement with, and barriers to, implementation of their countries' NAP-AMR. The third section asked about antimicrobial use (AMU) practices in their country and awareness of antimicrobial rating systems and antimicrobial prescribing guidelines. The final section asked respondents to indicate their level of agreement with statements regarding AMU and AMR surveillance systems in their country using a four-point Likert scale, ranging from strongly agree to strongly disagree.

A pretest of the questionnaire was conducted (with a previous Fleming Fund Fellow) to assess its clarity, comprehensibility, and relevance through a “think aloud” approach. Minor amendments were made to question format. Survey participants were recruited via email. Electronic survey responses were collected using a commercial survey instrument (Qualtrics [Provo, UT, USA]) and were anonymous. The survey was available from October to November 2024. Two reminders were sent to participants.

Descriptive statistics were computed with percentages being reported as the proportion of the total respondents answering a given question. Analyses were completed using functions within Excel and IBM SPSS (version 29.0.0.0).

This research was approved by the Human Research Ethics Committee of The University of Melbourne (Reference: 2024–30,443–58,383-3).

## 3 Results

In total, 102 participants, were invited. Responses were received from 82 participants (80 % response rate). Responses from 12 participants were excluded as less than 80 % of questions were completed.

Each country had at least ten responses in total, with participants from both the human and animal health sectors. Only two countries had responses from environmental representatives. The majority (60 %) of participants had more than 8 years of experience in their profession (Table 1).

**Table 1:** Demographic characteristics of the participants.

Demographic Characteristics		Frequency (n)	Percent (%)
Country	Nepal	18	26
	Bhutan	16	23
	Pakistan	15	21
	Papua New Guinea	11	16
	Timor-Leste	10	14
Field	Human Health	37	53
	Animal Health	28	40
	Environmental Health and/or Food Production and/or Food Safety	5	7
Experience	Less than 3 years	9	13
	3 to 5 years	8	11
	6 to 8 years	11	16
	More than 8 years	42	60
Fellowship Status	Current Fleming Fellow	36	51
	Former Fleming Fellow	34	49

Most (94 %) participants were aware their country had published a NAP-AMR. The majority of participants found the language of the NAP-AMR to be clear and easy to understand, with 98 % agreeing on its clarity. Similarly, 97 % participants found the document to be well-organized, with clear sections and a logical flow. Also, 58 % reported the NAP-AMR was accessible to stakeholders in their country in both printed and online formats, 28 % said it was only available online, and 14 % indicated it was only available in print. However, reporting was inconsistent between some participants from the same country. Participants were asked about the focus of NAP-AMR in tackling AMR across the human health, animal health, and environmental sectors. Participants believed their NAP-AMR focused on tackling AMR in the human health sector (78 % comprehensively, 22 % moderately) and animal health sector (41 % comprehensively, 52 % moderately) while there was less focus (45 % minimal 8 % not at all) on the environmental health sector. Typical objectives of NAP-AMR and participants' perceptions of progress toward their implementation are presented in Table 2.

**Table 2:** Typical national action plan on antimicrobial resistance objectives and participants' perceptions of progress toward implementation.

Typical national action plan on antimicrobial resistance objectives	Participants' perceptions on progress	Related figures
1. To improve awareness and understanding of antimicrobial resistance through effective communication, education and training	Doctors and veterinarians: well-aware in AMR, Policymakers: moderately aware Public, farmers, schoolchildren, and media: poor awareness.	Fig. 2
2. To strengthen the knowledge and evidence base through surveillance and research	Human and animal: AMU and AMR surveillance systems are established Environmental: AMR surveillance not established	Fig. 5
3. To reduce the incidence of infection through effective sanitation, hygiene and infection prevention measures	Not assessed	NA
4. To optimize the use of antimicrobial medicines in human and animal health	Human: Antimicrobial use comparatively well-managed Animal: Antimicrobial use moderately well managed Environmental: Antimicrobial use poorly managed	Fig. 3, Fig. 4
5. To develop the economic case for sustainable investment that takes account of the needs of all countries and to increase investment in new medicines, diagnostic tools, vaccines and other interventions.	Not assessed	NA

**Fig. 1:**
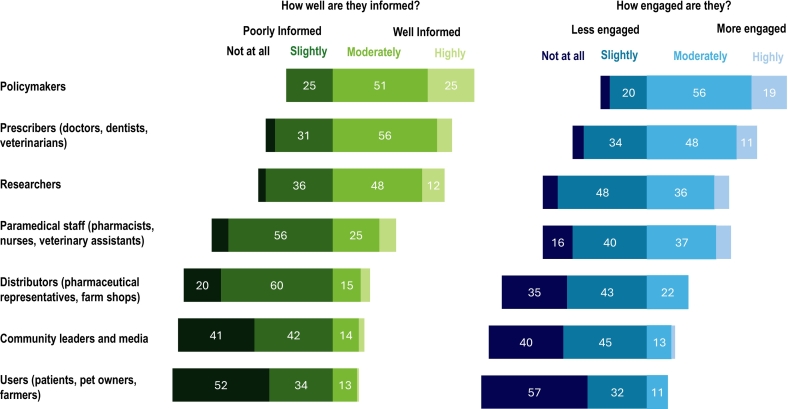
Participants' perceptions (*n* = 70) of stakeholder knowledge of National Action Plans on antimicrobial resistance in their country, and how actively these groups were engaged in implementing the National Action Plans on antimicrobial resistance. (Data labels omitted where values are less than 10 %).

There was considerable variation in participants' responses regarding how different stakeholder groups were informed and how they engaged in implementing the responsibilities outlined in the NAP-AMR within their work or practices. Stakeholder groups perceived to be well informed tended to also be seen to engage more actively. The well-informed stakeholder categories identified by participants included policymakers (25 % highly, 51 % moderately) and prescribers (8 % highly, 56 % moderately). Simultaneously, participants believed that policymakers (19 % highly, 56 % moderately) and prescribers (11 % highly, 48 % moderately) were more engaged with the implementation of NAP-AMR. In contrast, antibiotic distributors (20 % not at all, 60 % slightly) and users (52 % not at all, 34 % slightly) were identified as poorly informed stakeholders, while distributors (35 % not at all, 43 slightly) and users (57 % not at all, 32 % slightly) were also identified as less engaged stakeholders (Fig. 1).

**Fig. 2:**
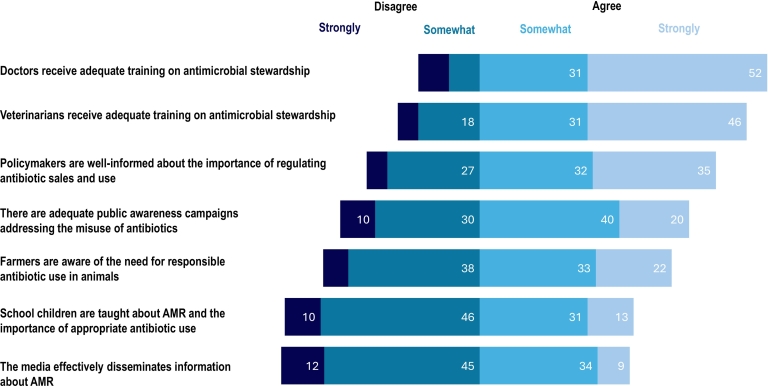
Participants' perceptions (n = 70) of awareness and training programmes on antimicrobial resistance in their country. (Data labels omitted where values are less than 10 %).

Most respondents believed that doctors (31 % somewhat or 52 % strongly agreed) and veterinarians (31 % somewhat and 46 % strongly agreed) received adequate training on antimicrobial stewardship. However, 33 % of participants disagreed (6 % strongly, 26 % somewhat) that policymakers were well-informed about the importance of regulating antibiotic sales and use. More than half of the participants disagreed (12 % strongly, 45 % somewhat) that the media effectively disseminates information about AMR (Fig. 2).

**Fig. 3:**
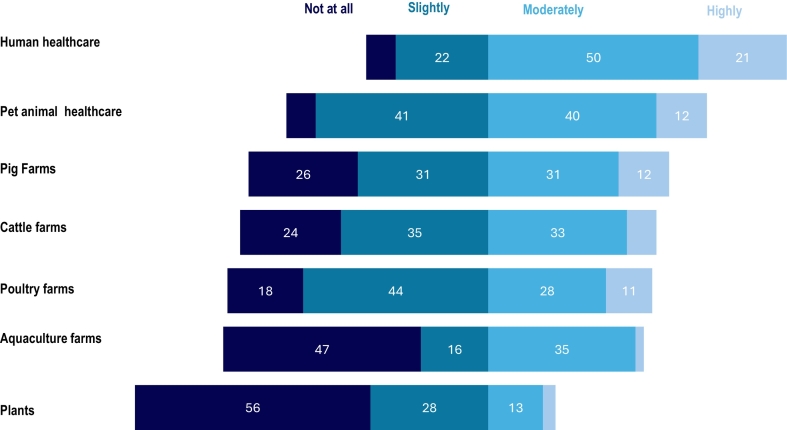
Participants' beliefs (n = 70) about the extent to which antimicrobials are used appropriately in human health, animal health, agriculture and aquaculture. (Data labels omitted where values are less than 10 %).

There was considerable variation in the perceived appropriateness of antimicrobial use (Fig. 3). Nearly three out of four participants believed that antimicrobials were appropriately used in the human healthcare sector (50 % moderately, 21 % highly). Participants believed that antimicrobials were used more appropriately (40 % moderately, 12 % highly) in the pet healthcare compared to pig (31 % moderately, 12 % highly), cattle (33 % moderately, 8 % highly) and poultry farming (28 %moderately, 11 % highly). Additionally, participants perceived that antimicrobials were not used appropriately at all in aquaculture farms (47 %) and the plant sector (56 %).

Participants reported that systemic antimicrobials (oral or injectable agents) were often accessed without a prescription. Specifically, 60 % of participants believed that systemic antimicrobials were typically obtained without a prescription for livestock, 42 % for pets, and 37 % for humans***.*** There was variation between countries in who prescribes antimicrobials, with some regions restricting prescribing to licensed medical professionals (e.g., doctors and veterinarians) and others where paramedical professionals played a role in antimicrobial prescribing.

**Fig. 4:**
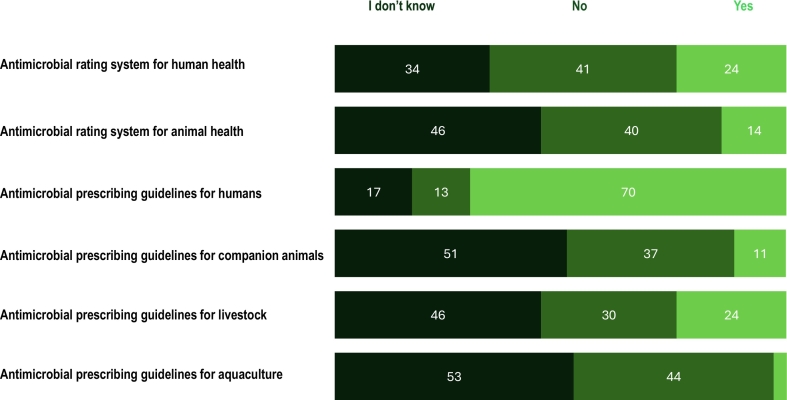
Participants' awareness (n = 70) of the availability of country-specific antimicrobial rating systems and antimicrobial prescribing guidelines in their country. (Data labels omitted where values are less than 10 %).

With regard to awareness of antimicrobial importance rating systems, the WHO Critically Important Antimicrobials for Human Medicine had the highest awareness (79 %), followed by the WOAH list of antimicrobial agents of veterinary importance (40 %). Approximately one-third (33 %) of participants were aware of both rating systems***.*** Participants' awareness of the availability of country-specific antimicrobial rating systems and antimicrobial prescribing guidelines were explored (Fig. 4). Nearly all participants believed that their country needed local antimicrobial prescribing guidelines for humans (94 %) and animals (86 %) but only, 70 % stated that antimicrobial prescribing guidelines were available for human health, while more than half of the participants did not know the availability of prescribing guidelines in the animal sector. Participants had varying levels of awareness regarding the existence of surveillance systems for antimicrobial resistance and antimicrobial use, with different participants within the same country having different understandings. The respondents reported that AMR surveillance systems exist in both human (81 %) and animal (51 %) sectors, whereas AMU surveillance systems were less common in both sectors (human 59 % and animal 33 %). In contrast, only a minority of the participants reported the presence of AMR (44 %) or AMU (42 %) surveillance systems in the environmental health sector. Furthermore, 39 % of participants reported that they didn't know whether AMU and AMR surveillance systems existed in the environmental sector.

**Fig. 5:**
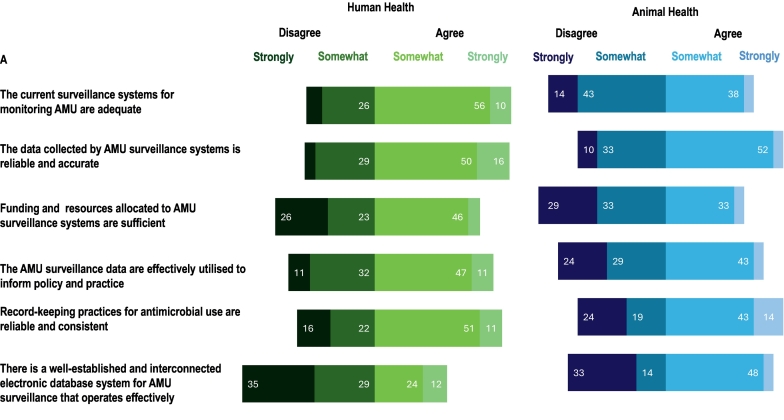

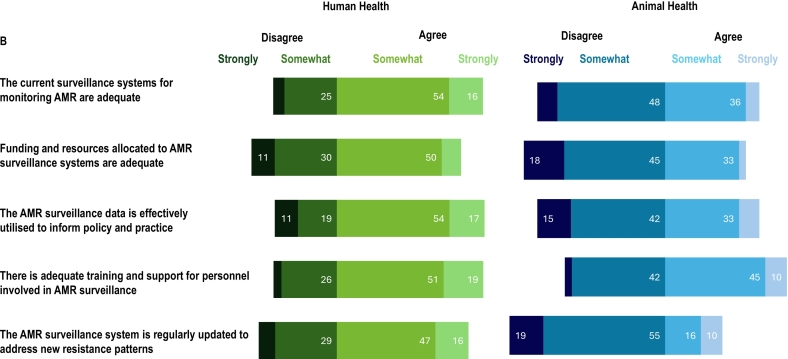
Participants' perceptions (n = 70) of surveillance systems in their country for (A) antimicrobial use (AMU) and (B) antimicrobial resistance (AMR) in the human and animal health sectors. (Data labels omitted where values are less than 10 %).

Participants were asked about the collaboration between the human, animal, and environmental sectors in AMR and AMR surveillance activities in their country. A small proportion of participants (7 % AMR, 4 % AMU) rated collaboration between all three sectors as excellent, while fewer than one-quarter of participants (23 % AMR, 18 % AMU) noted good collaboration between the human and animal sectors but not with the environmental sector. The most common response was that some collaboration existed between all three sectors in AMR surveillance (65 %) and AMU surveillance (47 %), although it remained limited and uncoordinated. Collaboration was reported to be absent between the three sectors in AMR and AMR surveillance by 4 % and 30 % of participants respectively. More than half of the participants agreed that both the current AMR (54 % somewhat, 16 % strongly) and AMU (56 % somewhat, 10 % strongly) surveillance systems were adequate for the human health sector (Fig. 5). In contrast, they disagreed with the statement for both AMR (48 % somewhat, 9 % strongly) and AMU (43 % somewhat, 14 % strongly) regarding the animal health sector. Nearly three-quarters of the participants agreed (54 % somewhat, 17 % strongly) that AMR surveillance in human sector data was effectively utilised to inform policy and practice. However, participants disagreed with that statement regarding both AMR (15 % somewhat, 42 % strongly) and AMU (29 % somewhat, 24 % strongly) surveillance systems in the animal health sector.

## 4 Discussion

This study reports perspectives of current NAP-AMR implementation among relevant professionals in the Asia-Pacific region for the first time. The study identified gaps in the implementation of NAP-AMR, and overall findings suggest the importance of implementing a transparent and strategic NAP-AMR monitoring and evaluation framework.

Our study reveals that the NAP-AMR implementation of the human health domain is relatively more advanced. In contrast, the environmental domain lags behind the other two domains, and there was broad recognition that NAP-AMR focus on the environmental sector is inadequate. This is unsurprising as insufficient governance of AMR in the environmental health sector was reported globally in both LMICs and HICs [21,22]. There could be several country-specific and universal reasons behind this inadequate governance in the environmental health domain [23,24]. Primarily, the involvement of international organizations like UNEP in the process of tackling and monitoring AMR came after the efforts of the WHO, WOAH and FAO [25]. Secondly, solely relying on government environment departments to tackle AMR in environmental sectors was identified as ineffective, as there is overlap with animal industries and pharmaceutical manufacturing. Hence, a network governance approach is recommended to tackle AMR in the environment, emphasising a shift from sector-specific NAP-AMR actions to shared responsibilities and collaborative goals across sectors [26,27].

The Quadripartite AMR country self-assessment survey (TrACSS), jointly administered by FAO, OIE, WHO and UNEP, aims to monitor progress in the implementation of the NAP-AMR [28,29]. Similarly, the Global Survey of Experts on Antimicrobial Resistance (GSEAR), assessed AMS and national implementation measures currently in place for optimizing antimicrobial use and slowing the spread of AMR in human medicine in LMICs [30]. However, the findings of our study and GSEAR showed a substantial amount of disagreement with TrACSS. TrACSS responses are generated through a self-assessment survey, and each country submits a consolidated official response. However, having one response per country may not accurately reflect the varying progress across different regions within that country. This could result in overestimated strengths or under-reported weaknesses. Furthermore, the perspectives of professionals who are responsible for implementing NAPs are important to identify gaps and challenges in implementation.

Knowledge gaps regarding AMR and AMU have been reported in different aspects worldwide and across a range of stakeholders [31,32]. However, in this study, participants believed that prescribers had adequate training and awareness of AMR. However, according to the TrACSS 2024, these countries failed to achieve the recommended level. This suggests a discordance between country and global expectations of knowledge and awareness that should be addressed. Importantly, one-third of participants disagreed that policymakers are well-informed about the importance of regulating antibiotic sales and use. This gap should be addressed urgently since all policymakers must be aware of the context in order to improve and implement effective policies.

According to the TrCASS 2024 report, only 39 % of HICs have implemented comprehensive AMU surveillance systems, while the majority of LMICs currently cannot monitor total antimicrobial sales at the national level [28]. None of the countries in this study effectively linked AMR data with antimicrobial consumption/use data for human health, although it should be noted that only 28 % of HICs achieved this goal. Surprisingly, over half of the participants perceived AMR and AMU surveillance as adequate in the human health sector, possibly due to poor awareness of expected standards. Perceptions regarding animal and environmental surveillance aligned with TrCASS 2024 findings [28]. Weakly integrated AMR and AMU surveillance systems were identified by the participants in this study. Lack of harmonisation in laboratory methods and interpretive criteria across surveillance components, within and between sectors, were identified as some of the main barriers to implementing an integrated AMR surveillance system, an issue also identified in HIC [33]. A study conducted in England found that poor data transparency and sharing between sectors were barriers to implementing integrated surveillance. Further, regardless of the importance, certain stakeholders chose not to share some sensitive data related to farms, creating barriers to integrated surveillance [34]. Further, the ineffective or complete absence of data sharing with policymakers has been identified in many NAP-AMR activities worldwide, and it also affects the translation of research results into policies and programs [13]. Since data sharing within sectors and between sectors and countries are important factors of the One Health approach and a global AMR response, further study is needed to identify drivers for data sharing and transparency.

Another challenge is the effective utilisation of data. Our participants disagreed that both AMU and AMR surveillance data are effectively utilised to inform policy and practice. Even when research work is technically sound, policymakers may find it too theoretical, inaccessible, or poorly timed [35,36]. There is a critical need for researchers to work with policymakers to strengthen evidence-based policy development as this is fundamental in building an effective policy framework for tackling AMR.

Unrestricted access to antimicrobials was identified as an issue in this study. Laws or regulations governing the prescription and sale of antimicrobials for human use are present in 84 % of LMICs, including all the countries in the current study [28]. While many countries (51 %) have policies regulating the over-the-counter sale of protected antibiotics, nearly 74 % still allowed antibiotics to be obtained without a prescription [30]. Counterfeit or substandard antimicrobials add to the problem [37]. We identified persistent perception of inappropriate use of antibiotics in all sectors. Due to socio-economic and political complexities of antimicrobial use and distribution, we recommend developing and revising policies through an evidence-informed policy approach. Evidence-informed policy considers scientific evidence but also incorporates values, public opinion, and political realities [38,39]. Country-specific antimicrobial prescribing guidelines, particularly for the animal health sectors, are also needed, since these are lacking in the settings studied. Antimicrobial prescribing guidelines promote judicious use of antimicrobials [40].

Primary limitations of this study was that it had a small sample size. For this reason, analysis of associations of perceptions with participants' demographics were not possible without affecting participant anonymity. There are many professionals relevant for NAP-AMR implementation in these countries and their perceptions may differ from the study population, which was a selected subset of these professionals. Another key limitation of this study is that the results of this study based on participants' personal perceptions, which may be influenced by subjective bias and therefore may not fully represent the broader reality of the current situation. Also, limited knowledge of NAP-AMR among participants may have affected their ability to assess the degree of implementation, which was identified as a limitation of this study. Further, a structured direct comparison with all the objectives in the NAPs was not possible due to the exploratory nature of the survey questions and the variability of the specific objectives in the NAPs across the countries.

The new findings from this study highlight expert perspectives on NAP-AMR implementation progress. Barriers to implementation included stakeholder engagement and awareness, lack of surveillance from all One Health domains and challenges related to optimizing antimicrobial usage. We recommend implementing systematic and transparent monitoring and evaluation frameworks across multiple One Health sectors [41]. Co-designing the framework by incorporating the perspectives of a broad range of stakeholders responsible for tackling AMR should be considered for the establishment of such a framework. Further research should explore the unique challenges encountered with NAP-AMR implementation under a One Health framework, and its links to social, cultural and political situations. Given the disagreement between individual professionals' perspectives and the TrACSS, it is important to continue to evaluate both perceptions to gain a broad understanding of implementation and challenges faced. A comprehensive evaluation process would help identify gaps in AMR strategies, improve policy implementation, and enable more effective tracking of progress toward global AMR goals. Furthermore, it could support better communication among stakeholders, including government agencies, healthcare professionals, and international organizations.

## CRediT authorship contribution statement

**Yasodhara D. Gunasekara:** Writing – original draft, Visualization, Validation, Software, Methodology, Formal analysis, Data curation, Conceptualization. **Kirsten E. Bailey:** Writing – review & editing, Visualization, Supervision, Software, Resources, Project administration, Methodology, Investigation, Conceptualization. **Ri O. Scarborough:** Writing – review & editing, Visualization, Software, Methodology, Data curation, Conceptualization. **Anna E. Sri:** Writing – review & editing, Data curation. **Mauricio J.C. Coppo:** Writing – review & editing, Supervision, Project administration, Investigation, Data curation. **James R. Gilkerson:** Writing – review & editing, Supervision, Resources. **Glenn F. Browning:** Writing – review & editing, Supervision, Resources, Project administration, Investigation, Funding acquisition, Conceptualization. **Laura Y. Hardefeldt:** Writing – review & editing, Validation, Supervision, Resources, Project administration, Methodology, Investigation, Funding acquisition, Data curation, Conceptualization.

## Funding

The 10.13039/501100000923Australian Research Council funded this study through the Discovery Early Career Research Award program (DE200100030) awarded to L.Y.H. and YDG is funded by the 10.13039/501100000923Australian Research Council (DE200100030).

## Declaration of competing interest

The authors declare that they have no known competing financial interests or personal relationships that could have appeared to influence the work reported in this paper.

## Data Availability

The data that has been used is confidential.

## Appendix A Supplementary data

Supplementary material Image 1

## References

[1] Transforming our world: the 2030 Agenda for Sustainable Development | Department of Economic and Social Affairs (2025). https://sdgs.un.org/2030agenda.

[2] amr-factsheet.pdf (2025). https://www.who.int/docs/default-source/antimicrobial-resistance/amr-factsheet.pdf.

[3] Aslam B., Asghar R., Muzammil S., Shafique M., Siddique A.B., Khurshid M., Ijaz M., Rasool M.H., Chaudhry T.H., Aamir A., Baloch Z. (2024). AMR and sustainable development goals: at a crossroads. Glob. Health.

[4] Sulis G., Sayood S., Gandra S. (2022). Antimicrobial resistance in low- and middle-income countries: current status and future directions. Expert Rev. Anti-Infect. Ther..

[5] Gunasekara Y., Kottawatta S., Nisansala T., Silva-Fletcher A., Kalupahana R., Vithanage M., Prasad M.N.V. (2023). One Health.

[6] Gunasekara Y.D., Kottawatta S.A., Nisansala T., Wijewickrama I.J.B., Basnayake Y.I., Silva-Fletcher A., Kalupahana R.S. (2024). Antibiotic resistance through the lens of One Health: a study from an urban and a rural area in Sri Lanka. Zoonoses Public Health.

[7] Hardefeldt L.Y., Thursky K. (2024). One Health antimicrobial resistance: stewardship in Australia. Microbiol. Aust..

[8] World Health Organization (2015). https://iris.who.int/handle/10665/193736.

[9] (2025). Implementing the Global Action Plan on Antimicrobial Resistance: First Quadripartite Biennial Report. https://www.who.int/publications/i/item/9789240074668.

[10] Patel J., Harant A., Fernandes G., Mwamelo A.J., Hein W., Dekker D., Sridhar D. (2023). Measuring the global response to antimicrobial resistance, 2020–21: a systematic governance analysis of 114 countries. Lancet Infect. Dis..

[11] Library of national action plans (2025). https://www.who.int/teams/surveillance-prevention-control-AMR/national-action-plan-monitoring-evaluation/library-of-national-action-plans.

[12] Willemsen A., Reid S., Assefa Y. (2022). A review of national action plans on antimicrobial resistance: strengths and weaknesses. Antimicrob. Resist. Infect. Control.

[13] Charani E., Mendelson M., Pallett S.J.C., Ahmad R., Mpundu M., Mbamalu O., Bonaconsa C., Nampoothiri V., Singh S., Peiffer-Smadja N., Anton-Vazquez V., Moore L.S.P., Schouten J., Kostyanev T., Vlahović-Palčevski V., Kofteridis D., Corrêa J.S., Holmes A.H. (2023). An analysis of existing national action plans for antimicrobial resistance—gaps and opportunities in strategies optimising antibiotic use in human populations. Lancet Glob. Health.

[14] W.H. Organization (2022).

[15] Gunasekara Y.D., Kinnison T., Kottawatta S.A., Kalupahana R.S., Silva-Fletcher A. (2022). Exploring barriers to one health antimicrobial stewardship in Sri Lanka: a qualitative study among healthcare professionals. Antibiotics.

[16] Gunasekera Y.D., Kinnison T., Kottawatta S.A., Silva-Fletcher A., Kalupahana R.S. (2022). Misconceptions of antibiotics as a potential explanation for their misuse. A survey of the general public in a rural and urban community in Sri Lanka. Antibiotics.

[17] Saman A., Chaudhry M., Ijaz M., Shaukat W., Zaheer M.U., Mateus A., Rehman A. (2023). Assessment of knowledge, perception, practices and drivers of antimicrobial resistance and antimicrobial usage among veterinarians in Pakistan. Prev. Vet. Med..

[18] Rijal K.R., Banjara M.R., Dhungel B., Kafle S., Gautam K., Ghimire B., Ghimire P., Dhungel S., Adhikari N., Shrestha U.T., Sunuwar D.R., Adhikari B., Ghimire P. (2021). Use of antimicrobials and antimicrobial resistance in Nepal: a nationwide survey. Sci. Rep..

[19] Naghavi M., Vollset S.E., Ikuta K.S., Swetschinski L.R., Gray A.P., Wool E.E., Aguilar G. Robles, Mestrovic T., Smith G., Han C., Hsu R.L., Chalek J., Araki D.T., Chung E., Raggi C., Hayoon A. Gershberg, Weaver N. Davis, Lindstedt P.A., Smith A.E., Altay U., Bhattacharjee N.V., Giannakis K., Fell F., McManigal B., Ekapirat N., Mendes J.A., Runghien T., Srimokla O., Abdelkader A., Abd-Elsalam S., Aboagye R.G., Abolhassani H., Abualruz H., Abubakar U., Abukhadijah H.J., Aburuz S., Abu-Zaid A., Achalapong S., Addo I.Y., Adekanmbi V., Adeyeoluwa T.E., Adnani Q.E.S., Adzigbli L.A., Afzal M.S., Afzal S., Agodi A., Ahlstrom A.J., Ahmad A., Ahmad S., Ahmad T., Ahmadi A., Ahmed A., Ahmed H., Ahmed I., Ahmed M., Ahmed S., Ahmed S.A., Akkaif M.A., Al Awaidy S., Al Thaher Y., Alalalmeh S.O., AlBataineh M.T., Aldhaleei W.A., Al-Gheethi A.A.S., Alhaji N.B., Ali A., Ali L., Ali S.S., Ali W., Allel K., Al-Marwani S., Alrawashdeh A., Altaf A., Al-Tammemi A.B., Al-Tawfiq J.A., Alzoubi K.H., Al-Zyoud W.A., Amos B., Amuasi J.H., Ancuceanu R., Andrews J.R., Anil A., Anuoluwa I.A., Anvari S., Anyasodor A.E., Apostol G.L.C., Arabloo J., Arafat M., Aravkin A.Y., Areda D., Aremu A., Artamonov A.A., Ashley E.A., Asika M.O., Athari S.S., Atout M.M.W., Awoke T., Azadnajafabad S., Azam J.M., Aziz S., Azzam A.Y., Babaei M., Babin F.-X., Badar M., Baig A.A., Bajcetic M., Baker S., Bardhan M., Barqawi H.J., Basharat Z., Basiru A., Bastard M., Basu S., Bayleyegn N.S., Belete M.A., Bello O.O., Beloukas A., Berkley J.A., Bhagavathula A.S., Bhaskar S., Bhuyan S.S., Bielicki J.A., Briko N.I., Brown C.S., Browne A.J., Buonsenso D., Bustanji Y., Carvalheiro C.G., Castañeda-Orjuela C.A., Cenderadewi M., Chadwick J., Chakraborty S., Chandika R.M., Chandy S., Chansamouth V., Chattu V.K., Chaudhary A.A., Ching P.R., Chopra H., Chowdhury F.R., Chu D.-T., Chutiyami M., Cruz-Martins N., da Silva A.G., Dadras O., Dai X., Darcho S.D., Das S., De la Hoz F.P., Dekker D.M., Dhama K., Diaz D., Dickson B.F.R., Djorie S.G., Dodangeh M., Dohare S., Dokova K.G., Doshi O.P., Dowou R.K., Dsouza H.L., Dunachie S.J., Dziedzic A.M., Eckmanns T., Ed-Dra A., Eftekharimehrabad A., Ekundayo T.C., El Sayed I., Elhadi M., El-Huneidi W., Elias C., Ellis S.J., Elsheikh R., Elsohaby I., Eltaha C., Eshrati B., Eslami M., Eyre D.W., Fadaka A.O., Fagbamigbe A.F., Fahim A., Fakhri-Demeshghieh A., Fasina F.O., Fasina M.M., Fatehizadeh A., Feasey N.A., Feizkhah A., Fekadu G., Fischer F., Fitriana I., Forrest K.M., Rodrigues C. Fortuna, Fuller J.E., Gadanya M.A., Gajdács M., Gandhi A.P., Garcia-Gallo E.E., Garrett D.O., Gautam R.K., Gebregergis M.W., Gebrehiwot M., Gebremeskel T.G., Geffers C., Georgalis L., Ghazy R.M., Golechha M., Golinelli D., Gordon M., Gulati S., Gupta R.D., Gupta S., Gupta V.K., Habteyohannes A.D., Haller S., Harapan H., Harrison M.L., Hasaballah A.I., Hasan I., Hasan R.S., Hasani H., Haselbeck A.H., Hasnain M.S., Hassan I.I., Hassan S., Tabatabaei M.S. Hassan Zadeh, Hayat K., He J., Hegazi O.E., Heidari M., Hezam K., Holla R., Holm M., Hopkins H., Hossain M.M., Hosseinzadeh M., Hostiuc S., Hussein N.R., Huy L.D., Ibáñez-Prada E.D., Ikiroma A., Ilic I.M., Islam S.M.S., Ismail F., Ismail N.E., Iwu C.D., Iwu-Jaja C.J., Jafarzadeh A., Jaiteh F., Yengejeh R. Jalilzadeh, Jamora R.D.G., Javidnia J., Jawaid T., Jenney A.W.J., Jeon H.J., Jokar M., Jomehzadeh N., Joo T., Joseph N., Kamal Z., Kanmodi K.K., Kantar R.S., Kapisi J.A., Karaye I.M., Khader Y.S., Khajuria H., Khalid N., Khamesipour F., Khan A., Khan M.J., Khan M.T., Khanal V., Khidri F.F., Khubchandani J., Khusuwan S., Kim M.S., Kisa A., Korshunov V.A., Krapp F., Krumkamp R., Kuddus M., Kulimbet M., Kumar D., Kumaran E.A.P., Kuttikkattu A., Kyu H.H., Landires I., Lawal B.K., Le T.T.T., Lederer I.M., Lee M., Lee S.W., Lepape A., Lerango T.L., Ligade V.S., Lim C., Lim S.S., Limenh L.W., Liu C., Liu X., Liu X., Loftus M.J., Amin H.I.M., Maass K.L., Maharaj S.B., Mahmoud M.A., Maikanti-Charalampous P., Makram O.M., Malhotra K., Malik A.A., Mandilara G.D., Marks F., Martinez-Guerra B.A., Martorell M., Masoumi-Asl H., Mathioudakis A.G., May J., McHugh T.A., Meiring J., Meles H.N., Melese A., Melese E.B., Minervini G., Mohamed N.S., Mohammed S., Mohan S., Mokdad A.H., Monasta L., Ghalibaf A. Moodi, Moore C.E., Moradi Y., Mossialos E., Mougin V., Mukoro G.D., Mulita F., Muller-Pebody B., Murillo-Zamora E., Musa S., Musicha P., Musila L.A., Muthupandian S., Nagarajan A.J., Naghavi P., Nainu F., Nair T.S., Najmuldeen H.H.R., Natto Z.S., Nauman J., Nayak B.P., Nchanji G.T., Ndishimye P., Negoi I., Negoi R.I., Nejadghaderi S.A., Nguyen Q.P., Noman E.A., Nwakanma D.C., O’Brien S., Ochoa T.J., Odetokun I.A., Ogundijo O.A., Ojo-Akosile T.R., Okeke S.R., Okonji O.C., Olagunju A.T., Olivas-Martinez A., Olorukooba A.A., Olwoch P., Onyedibe K.I., Ortiz-Brizuela E., Osuolale O., Ounchanum P., Oyeyemi O.T., A M.P.P., Paredes J.L., Parikh R.R., Patel J., Patil S., Pawar S., Peleg A.Y., Peprah P., Perdigão J., Perrone C., Petcu I.-R., Phommasone K., Piracha Z.Z., Poddighe D., Pollard A.J., Poluru R., Ponce-De-Leon A., Puvvula J., Qamar F.N., Qasim N.H., Rafai C.D., Raghav P., Rahbarnia L., Rahim F., Rahimi-Movaghar V., Rahman M., Rahman M.A., Ramadan H., Ramasamy S.K., Ramesh P.S., Ramteke P.W., Rana R.K., Rani U., Rashidi M.-M., Rathish D., Rattanavong S., Rawaf S., Redwan E.M.M., Reyes L.F., Roberts T., Robotham J.V., Rosenthal V.D., Ross A.G., Roy N., Rudd K.E., Sabet C.J., Saddik B.A., Saeb M.R., Saeed U., Moghaddam S. Saeedi, Saengchan W., Safaei M., Saghazadeh A., Sharif-Askari N. Saheb, Sahebkar A., Sahoo S.S., Sahu M., Saki M., Salam N., Saleem Z., Saleh M.A., Samodra Y.L., Samy A.M., Saravanan A., Satpathy M., Schumacher A.E., Sedighi M., Seekaew S., Shafie M., Shah P.A., Shahid S., Shahwan M.J., Shakoor S., Shalev N., Shamim M.A., Shamshirgaran M.A., Shamsi A., Sharifan A., Shastry R.P., Shetty M., Shittu A., Shrestha S., Siddig E.E., Sideroglou T., Sifuentes-Osornio J., Silva L.M.L.R., Simões E.A.F., Simpson A.J.H., Singh A., Singh S., Sinto R., Soliman S.S.M., Soraneh S., Stoesser N., Stoeva T.Z., Swain C.K., Szarpak L., Ty S.S., Tabatabai S., Tabche C., Taha Z.M.-A., Tan K.-K., Tasak N., Tat N.Y., Thaiprakong A., Thangaraju P., Tigoi C.C., Tiwari K., Tovani-Palone M.R., Tran T.H., Tumurkhuu M., Turner P., Udoakang A.J., Udoh A., Ullah N., Ullah S., Vaithinathan A.G., Valenti M., Vos T., Vu H.T.L., Waheed Y., Walker A.S., Walson J.L., Wangrangsimakul T., Weerakoon K.G., Wertheim H.F.L., Williams P.C.M., Wolde A.A., Wozniak T.M., Wu F., Wu Z., Yadav M.K.K., Yaghoubi S., Yahaya Z.S., Yarahmadi A., Yezli S., Yismaw Y.E., Yon D.K., Yuan C.-W., Yusuf H., Zakham F., Zamagni G., Zhang H., Zhang Z.-J., Zielińska M., Zumla A., Zyoud S.H.H., Zyoud S.H., Hay S.I., Stergachis A., Sartorius B., Cooper B.S., Dolecek C., Murray C.J.L. (2024). Global burden of bacterial antimicrobial resistance 1990–2021: a systematic analysis with forecasts to 2050. Lancet.

[20] Murray C.J.L., Ikuta K.S., Sharara F., Swetschinski L., Aguilar G.R., Gray A., Han C., Bisignano C., Rao P., Wool E., Johnson S.C., Browne A.J., Chipeta M.G., Fell F., Hackett S., Haines-Woodhouse G., Hamadani B.H.K., Kumaran E.A.P., McManigal B., Achalapong S., Agarwal R., Akech S., Albertson S., Amuasi J., Andrews J., Aravkin A., Ashley E., Babin F.-X., Bailey F., Baker S., Basnyat B., Bekker A., Bender R., Berkley J.A., Bethou A., Bielicki J., Boonkasidecha S., Bukosia J., Carvalheiro C., Castañeda-Orjuela C., Chansamouth V., Chaurasia S., Chiurchiù S., Chowdhury F., Donatien R.C., Cook A.J., Cooper B., Cressey T.R., Criollo-Mora E., Cunningham M., Darboe S., Day N.P.J., Luca M.D., Dokova K., Dramowski A., Dunachie S.J., Bich T.D., Eckmanns T., Eibach D., Emami A., Feasey N., Fisher-Pearson N., Forrest K., Garcia C., Garrett D., Gastmeier P., Giref A.Z., Greer R.C., Gupta V., Haller S., Haselbeck A., Hay S.I., Holm M., Hopkins S., Hsia Y., Iregbu K.C., Jacobs J., Jarovsky D., Javanmardi F., Jenney A.W.J., Khorana M., Khusuwan S., Kissoon N., Kobeissi E., Kostyanev T., Krapp F., Krumkamp R., Kumar A., Kyu H.H., Lim C., Lim K., Limmathurotsakul D., Loftus M.J., Lunn M., Ma J., Manoharan A., Marks F., May J., Mayxay M., Mturi N., Munera-Huertas T., Musicha P., Musila L.A., Mussi-Pinhata M.M., Naidu R.N., Nakamura T., Nanavati R., Nangia S., Newton P., Ngoun C., Novotney A., Nwakanma D., Obiero C.W., Ochoa T.J., Olivas-Martinez A., Olliaro P., Ooko E., Ortiz-Brizuela E., Ounchanum P., Pak G.D., Paredes J.L., Peleg A.Y., Perrone C., Phe T., Phommasone K., Plakkal N., Ponce-de-Leon A., Raad M., Ramdin T., Rattanavong S., Riddell A., Roberts T., Robotham J.V., Roca A., Rosenthal V.D., Rudd K.E., Russell N., Sader H.S., Saengchan W., Schnall J., Scott J.A.G., Seekaew S., Sharland M., Shivamallappa M., Sifuentes-Osornio J., Simpson A.J., Steenkeste N., Stewardson A.J., Stoeva T., Tasak N., Thaiprakong A., Thwaites G., Tigoi C., Turner C., Turner P., van Doorn H.R., Velaphi S., Vongpradith A., Vongsouvath M., Vu H., Walsh T., Walson J.L., Waner S., Wangrangsimakul T., Wannapinij P., Wozniak T., Sharma T.E.M.W.Y., Yu K.C., Zheng P., Sartorius B., Lopez A.D., Stergachis A., Moore C., Dolecek C., Naghavi M. (2022). Global burden of bacterial antimicrobial resistance in 2019: a systematic analysis. Lancet.

[21] Coque T., Graham D.W., Pruden A., So A., Topp E. (2025).

[22] Sabbatucci M., Ashiru-Oredope D., Barbier L., Bohin E., Bou-Antoun S., Brown C., Clarici A., Fuentes C., Goto T., Maraglino F., Morin J., Rönnefahrt I., Sanwidi A., Triggs-Hodge C., Vitiello A., Zovi A., Gelormini M., Wong D. Lo Fo (2024). Tracking progress on antimicrobial resistance by the quadripartite country self-assessment survey (TrACSS) in G7 countries, 2017–2023: opportunities and gaps. Pharmacol. Res..

[23] Green D.M., Desbois A.P., Elumalai P., Lakshmi S. (2025). Antimicrobial Resistance in Aquaculture and Aquatic Environments.

[24] Larsson D.G.J., Gaze W.H., Laxminarayan R., Topp E. (2023). AMR, One Health and the environment. Nat. Microbiol..

[25] (2025). One Health Joint Plan of Action (2022–2026): Working Together for the Health of Humans, Animals, Plants and the Environment. https://www.who.int/publications/i/item/9789240059139.

[26] Birgand G., Castro-Sánchez E., Hansen S., Gastmeier P., Lucet J.-C., Ferlie E., Holmes A., Ahmad R. (2018). Comparison of governance approaches for the control of antimicrobial resistance: analysis of three European countries. Antimicrob. Resist. Infect. Control.

[27] (2025). Fragmentation in One Health Policy and Practice Responses to Antimicrobial Resistance and the Salutary Value of Collaborative Humility | Social Theory & Health. http://dx.doi.org/10.1057/s41285-024-00209-2.

[28] (2025). Global Database for Tracking Antimicrobial Resistance (AMR) Country Self- Assessment Survey (TrACSS). https://new.amrcountryprogress.org/.

[29] (2025). Results from the 2024 Tracking Antimicrobial Resistance Country Self-Assessment Survey (TrACSS): Quadripartite Webinar. https://www.who.int/news-room/events/detail/2024/09/10/default-calendar/results-from-the-2024-tracking-antimicrobial-resistance-country-self-assessment-survey-(tracss)--quadripartite-webinar.

[30] Zay Ya K., Lambiris M.J., Levine G.A., Tediosi F., Fink G. (2024). Coverage of policies to improve antimicrobial stewardship in human medicine in low and middle income countries: results from the Global Survey of Experts on Antimicrobial Resistance. BMC Public Health.

[31] (2025). Exploring Barriers to One Health Antimicrobial Stewardship in Sri Lanka: A Qualitative Study among Healthcare Professionals. https://www.mdpi.com/2079-6382/11/7/968.

[32] Scarborough R.O., Bailey K.E., Sri A.E., Browning G.F., Hardefeldt L.Y. (2024). Seeking simplicity, navigating complexity: how veterinarians select an antimicrobial drug, dose, and duration for companion animals. J. Vet. Intern. Med..

[33] Hardefeldt L., Marenda M., Crabb H., Stevenson M., Gilkerson J., Billman-Jacobe H., Browning G. (2018). Antimicrobial susceptibility testing by Australian veterinary diagnostic laboratories. Aust. Vet. J..

[34] Bennani H., Cornelsen L., Stärk K.D.C., Häsler B. (2021). Evaluating integrated surveillance for antimicrobial use and resistance in England: a qualitative study. Front. Vet. Sci..

[35] Hunter D.J. (2009). Relationship between evidence and policy: a case of evidence-based policy or policy-based evidence?. Public Health.

[36] Kemm J. (2006). The limitations of ‘evidence-based’ public health. J. Eval. Clin. Pract..

[37] (2025). Substandard and Falsified Medical Products. https://www.who.int/news-room/fact-sheets/detail/substandard-and-falsified-medical-products.

[38] Fenna A. (2015). Public policy in the Australian journal of political science: a review. Aust. J. Polit. Sci..

[39] Fenna A. (2021). http://ebookcentral.proquest.com/lib/unimelb/detail.action?docID=6480981.

[40] Richards S., Bailey K.E., Scarborough R., Gilkerson J.R., Browning G.F., Hur B., Ierardo J., Awad M., Chay R., Hardefeldt L.Y. (2024). Cross-sectional evaluation of a large-scale antimicrobial stewardship trial in Australian companion animal practices. Vet. Rec..

[41] (2025). Guidance to Facilitate Monitoring and Evaluation for Antimicrobial Resistance National Action Plans. https://www.who.int/publications/i/item/9789240069763.

