# Supplementary material for: National action plan on antimicrobial resistance in selected Asia-Pacific low- and middle-income countries: Perspectives of One Health stakeholders
Source: One Health. 2025 Oct 30;21:101259. doi: 10.1016/j.onehlt.2025.101259 (PMC12637263; doi:10.1016/j.onehlt.2025.101259)
Supplement: Supplementary file 1 — Supplementary material [file mmc1.docx]

Antimicrobial Stewardship Status: Perspectives from Participants in Low- and Middle-Income Countries

Start of Block: Survey and consent form

Consent  Project: One Health Antimicrobial Stewardship in Low and Middle-Income Countries: Identifying Barriers and Facilitators for Effective Implementation **Project Supervisor:** Dr. Laura Hardefeldt Tel: +61 3 9035 3562 Email: laura.hardefeldt@unimelb.edu.au. **Additional Researchers:** Dr Kirsten Bailey Email: baileyk@unimelb.edu.au Prof Glenn Browning Email: glenfb@unimelb.edu.au Prof James Gilkerson Email: jrgilk@unimelb.edu.au Dr Mauricio Coppo Email: mjcoppo@unimelb.edu.au. Dr Ri Scarborough Email: ri.scarborough@unimelb.edu.au. Dr Anna Sri Email: anna.sri@student.unimelb.edu.au Dr Yasodhara Gunasekara Email: yasodhara.gunasekara@unimelb.edu.au Thank you for your interest in participating in this research project. We ask that you read important information about this project in the plain language statement Click here to read the plain language statement **Consent to participate in the survey:** 1. I consent to participate in this project, the details of which have been explained to me, and I have been provided with a written plain language statement. 2. I understand that the purpose of this research is to investigate existing Antimicrobial Stewardship programs and gather insights on the barriers and facilitators 3. I understand that my participation in this project is for research purposes only. 4. I acknowledge that the possible effects of participating in this research project have been explained to my satisfaction. 5. In this project, I will be required to complete a 15 to 20-minute online survey about antimicrobial stewardship in my country. 6. I understand that my participation is voluntary and that I am free to withdraw from this project anytime without explanation or prejudice. However, I understand I cannot withdraw any responses that I have provided since my responses will not be identified. 7. I understand that the data from this research will be stored at the University of Melbourne and will be destroyed 5 years after publication. 8. I have been informed that the confidentiality of the information I provide will be safeguarded; my data will be password protected and accessible only by the named researchers. 9. I understand that after I complete this consent form, it will be retained by the researcher. If you are willing to participate in the survey, please give your consent by clicking "Yes"

- Yes (1)
- No (2)

Skip To: End of Survey If Project: One Health Antimicrobial Stewardship in Low and Middle-Income Countries: Identifying Bar... = No

End of Block: Survey and consent form

Start of Block: Participants Demographics

Country **Please note that in this questionnaire, the animal health sector includes pet animals, livestock, and aquaculture.** In which country do you work?

- Bhutan (1)
- Nepal (2)
- Pakistan (3)
- Papua New Guinea (4)
- Timor-Leste (5)

Profession To which field does your job most closely align?

- Human Health (1)
- Animal Health (2)
- Environmental Health (3)
- Food Production and/or Food Safety (4)

Year in Role How many years have you been in your current role?

- Less than 3 years (1)
- 3 to 5 years (2)
- 6 to 8 years (3)
- More than 8 years (4)

Fellowship status What is your current status with the Fleming Fellowship program?

- Current Fleming Fellow (1)
- Former Fleming Fellow (Completed the program) (2)

End of Block: Participants Demographics

Start of Block: National Action Plan

1 Has your government produced a national action plan for tackling AMR?

- Yes (1)
- No (2)
- I'm not aware whether we have an action plan or not (3)

| Page Break |  |
| --- | --- |

Display This Question:

If Has your government produced a national action plan for tackling AMR? = Yes

2 What is the **focus** of your country's national action plan in tackling AMR across various sectors (human health, animal health, and the environment)?

|  | Comprehensively (4) | Moderately (3) | Minimally (2) | Not at all (1) |
| --- | --- | --- | --- | --- |
| Human health (1) |  |  |  |  |
| Animal health (2) |  |  |  |  |
| Environmental health (3) |  |  |  |  |

Display This Question:

If Has your government produced a national action plan for tackling AMR? = Yes

3 To what **extent** do you **agree** with the following statements about the **format** of the National Action Plan on AMR?

|  | Strongly agree (1) | Somewhat agree (2) | Somewhat disagree (4) | Strongly disagree (5) | I don't know (6) |
| --- | --- | --- | --- | --- | --- |
| The language of the National Action Plan is clear and easy to understand, ensuring accessibility to readers from various backgrounds (1) |  |  |  |  |  |
| The document is well-organized with clear sections and logical flow, making it easy to navigate and follow (2) |  |  |  |  |  |

Display This Question:

If Has your government produced a national action plan for tackling AMR? = Yes

4 How **accessible** is the National Action Plan on AMR to stakeholders in your country?

- Available online and in print (1)
- Available online only (2)
- Available in print only (3)
- Not available online or in print (4)
- I don't know (5)

| Page Break |  |
| --- | --- |

Display This Question:

If Has your government produced a national action plan for tackling AMR? = Yes

5 How **informed** are the following **stakeholder** groups about the National Action Plan on AMR in your country? Highly informed (aware of the plan and its details) Moderately informed (aware of the plan but not familiar with specifics) Slightly informed (aware of the plan’s existence but unaware of its content) Not informed (unaware of the plan)

|  | Highly informed (1) | Moderately informed (2) | Slightly informed (3) | Not informed (4) | I don't know (5) |
| --- | --- | --- | --- | --- | --- |
| Policymakers (1) |  |  |  |  |  |
| Prescribers (e.g. doctors, dentists, veterinarians) (2) |  |  |  |  |  |
| Paramedical staff (e.g. pharmacists, nurses, veterinary assistants) (3) |  |  |  |  |  |
| Distributors (e.g. pharmaceutical industry representatives, farm shop managers) (4) |  |  |  |  |  |
| Users (patients, pet owners, farmers) (5) |  |  |  |  |  |
| Community leaders and media (6) |  |  |  |  |  |
| Researchers (7) |  |  |  |  |  |

| Page Break |  |
| --- | --- |

Display This Question:

If Has your government produced a national action plan for tackling AMR? = Yes

6 How **actively** are the following **stakeholder** groups engaged with implementing the responsibilities outlined in the National Action Plan on AMR in their work or practices? Highly engaged (actively involved in implementing and fulfilling their responsibilities under the plan) Moderately engaged (some involvement in implementing the plan, but not consistent) Slightly engaged (minimal involvement or occasional reference to the plan in their work) Not engaged (no involvement with the plan)

|  | Highly engaged (1) | Moderately engaged (2) | Slightly engaged (3) | Not engaged (4) | I don't Know (5) |
| --- | --- | --- | --- | --- | --- |
| Policymakers (1) |  |  |  |  |  |
| Prescribers (e.g. doctors, dentists, veterinarians) (2) |  |  |  |  |  |
| Paramedical staff (e.g. pharmacists, nurses, veterinary assistants) (3) |  |  |  |  |  |
| Distributors (e.g. pharmaceutical industry representatives, farm shop managers) (4) |  |  |  |  |  |
| Users (patients, pet owners, farmers) (5) |  |  |  |  |  |
| Community leaders and media (6) |  |  |  |  |  |
| Researchers (7) |  |  |  |  |  |

| Page Break |  |
| --- | --- |

Display This Question:

If Has your government produced a national action plan for tackling AMR? = Yes

7 To what **extent** do you feel the current national action plan in your country is being **effectively** implemented to control AMR as intended?

- Not at all (1)
- To a small extent (2)
- To a moderate extent (3)
- To a great extent (4)
- I prefer not to answer (5)

| Page Break |  |
| --- | --- |

Display This Question:

If Has your government produced a national action plan for tackling AMR? = Yes

8 What is **stopping** effective implementation of the national action plan in your country?

|  | Not a Barrier (1) | A Barrier (2) | I don't know (3) |
| --- | --- | --- | --- |
| Insufficient funding (1) |  |  |  |
| Limited public awareness (2) |  |  |  |
| Resistance from healthcare professionals (4) |  |  |  |
| Lack of political commitment (5) |  |  |  |
| Insufficient collaboration and coordination between sectors (6) |  |  |  |
| Challenges in regulatory enforcement (7) |  |  |  |
| Inadequate surveillance and monitoring systems (8) |  |  |  |
| Income disparities and healthcare infrastructure challenges in rural or underserved areas (9) |  |  |  |
| Lack of professional expertise (10) |  |  |  |
| Lack of international cooperation and standards (11) |  |  |  |
| Limited access to affordable diagnostics (12) |  |  |  |
| Limited access to affordable treatments (13) |  |  |  |

| Page Break |  |
| --- | --- |

Display This Question:

If Has your government produced a national action plan for tackling AMR? = Yes

| 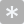 |
| --- |

9 Please **rank the top 3 barriers** affecting implementation of the national action plan to tackle AMR in your country. (Place number 1 for the most significant barrier, number 2 for the second most significant barrier, and number 3 for the third most significant barrier)

______ Insufficient funding (1)

______ Limited public awareness (2)

______ Resistance from healthcare professionals (4)

______ Lack of political commitment (5)

______ Insufficient collaboration and coordination between sectors (6)

______ Challenges in regulatory enforcement (7)

______ Inadequate surveillance and monitoring systems (8)

______ Income disparities and healthcare infrastructure challenges in rural or underserved areas (9)

______ Lack of professional expertise (10)

______ Lack of international cooperation and standards (11)

______ Limited access to affordable diagnostics (12)

______ Limited access to affordable treatments (13)

Display This Question:

If Has your government produced a national action plan for tackling AMR? = Yes

10 In your country, if you have additional barriers that are not mentioned above, please write them below.

________________________________________________________________

________________________________________________________________

________________________________________________________________

________________________________________________________________

________________________________________________________________

End of Block: National Action Plan

Start of Block: Prescribing Guidelines

11  Which antimicrobial **importance rating** systems are you aware of?

- WHO (World Health Organization) Critically important antimicrobials for human medicine (1)
- WOAH (World Organisation for Animal Health, previously OIE) List of antimicrobial agents of veterinary importance (3)
- I am not aware of any antimicrobial importance rating systems (5)

12 If you are aware of other antimicrobial rating systems that are not mentioned above, please write them below

________________________________________________________________

________________________________________________________________

________________________________________________________________

________________________________________________________________

________________________________________________________________

Display This Question:

If  Which antimicrobial importance rating systems are you aware of? = WHO (World Health Organization) Critically important antimicrobials for human medicine

Or  Which antimicrobial importance rating systems are you aware of? = WOAH (World Organisation for Animal Health, previously OIE) List of antimicrobial agents of veterinary importance

13 Does your country have its own (country-specific) antimicrobial **rating** system?

|  | Yes (1) | No (2) | I don't know (3) |
| --- | --- | --- | --- |
| Antimicrobial rating system for human health (1) |  |  |  |
| Antimicrobial rating system for animal health (2) |  |  |  |

| Page Break |  |
| --- | --- |

14 How do people in your country typically **access** systemic antimicrobials (e.g. oral/injectable amoxicillin) for the following species?

|  | With prescription (1) | Without prescription (2) | I don't know (3) |
| --- | --- | --- | --- |
| Livestock (1) |  |  |  |
| Companion animals (2) |  |  |  |
| Humans (3) |  |  |  |

15 Who can prescribe antimicrobials in your country? (select all that apply)

- Doctors (1)
- Nurses (2)
- Veterinarians (3)
- Para-veterinarians (4)
- Pharmacists (5)
- Dentists (6)

16 If anyone else can prescribe antimicrobials that is not mentioned above, please write it below.

________________________________________________________________

________________________________________________________________

________________________________________________________________

________________________________________________________________

________________________________________________________________

17 Does your country have its own (country-specific) antimicrobial prescribing **guidelines**?

|  | Yes (1) | No (2) | I don't know (3) |
| --- | --- | --- | --- |
| Antimicrobial Prescribing Guidelines for humans (1) |  |  |  |
| Antimicrobial Prescribing Guidelines for Companion animals (2) |  |  |  |
| Antimicrobial Prescribing Guidelines for livestock animals (3) |  |  |  |
| Antimicrobial Prescribing Guidelines for Aquaculture (4) |  |  |  |

18 Do you think it is **important** for your country to have your own **country-specific** antimicrobial prescribing **guidelines**?

|  | Highly Important (1) | Moderately Important (2) | Slightly Important (3) | Not Important (4) |
| --- | --- | --- | --- | --- |
| Antimicrobial Prescribing Guidelines for humans (1) |  |  |  |  |
| Antimicrobial Prescribing Guidelines for animals (2) |  |  |  |  |

19 Please indicate the **extent** to which you believe antimicrobials are being **used appropriately** in the following sectors in your country.

|  | Highly (5) | Moderately (3) | Slightly (4) | Not at all (1) | I don't know (6) |
| --- | --- | --- | --- | --- | --- |
| Human healthcare (1) |  |  |  |  |  |
| Pet animal healthcare (2) |  |  |  |  |  |
| Poultry farms (3) |  |  |  |  |  |
| Cattle farms (4) |  |  |  |  |  |
| Aquaculture farms (5) |  |  |  |  |  |
| Pig farms (6) |  |  |  |  |  |
| Plants (7) |  |  |  |  |  |

End of Block: Prescribing Guidelines

Start of Block: Surveillance systems

20 Does your country have an **AMR** **surveillance** system in the **human health** sector?

- Yes (2)
- No (3)
- I don't know (4)

Display This Question:

If Does your country have an AMR surveillance system in the human health sector? = Yes

21 How **strongly** do you agree or disagree with the following statements about the **adequacy** of AMR surveillance systems in the **human health sector** in your country?

|  | Strongly agree (1) | Somewhat agree (3) | Somewhat disagree (4) | Strongly disagree (5) | I don't know (6) |
| --- | --- | --- | --- | --- | --- |
| The current surveillance systems for monitoring AMR are adequate (1) |  |  |  |  |  |
| The funding and resources allocated to AMR surveillance systems are adequate (2) |  |  |  |  |  |
| The AMR surveillance data is effectively utilised to inform policy and practice (3) |  |  |  |  |  |
| There is adequate training and support for personnel involved in AMR surveillance (4) |  |  |  |  |  |
| The AMR surveillance system is regularly updated to address new resistance patterns (7) |  |  |  |  |  |

| Page Break |  |
| --- | --- |

22 Does your country have an **AMR** **surveillance** system in the **animal health** sector?

- Yes (1)
- No (2)
- I don't know (3)

Display This Question:

If Does your country have an AMR surveillance system in the animal health sector? = Yes

23 How **strongly** do you agree or disagree with the following statements about the **adequacy** of AMR surveillance systems in the **animal health sector** in your country?

|  | Strongly agree (1) | Somewhat agree (3) | Somewhat disagree (4) | Strongly disagree (5) | I don't know (6) |
| --- | --- | --- | --- | --- | --- |
| The current surveillance systems for monitoring AMR are adequate (1) |  |  |  |  |  |
| The funding and resources allocated to AMR surveillance systems are adequate (2) |  |  |  |  |  |
| The AMR surveillance data is effectively utilised to inform policy and practice (3) |  |  |  |  |  |
| There is adequate training and support for personnel involved in AMR surveillance (4) |  |  |  |  |  |
| The AMR surveillance system is regularly updated to address new resistance patterns (7) |  |  |  |  |  |

| Page Break |  |
| --- | --- |

24 Does your country have an **AMR** **surveillance** system in the **environmental health** sector?

- Yes (1)
- No (2)
- I don't know (3)

Display This Question:

If Does your country have an AMR surveillance system in the environmental health sector? = Yes

25 How **strongly** do you agree or disagree with the following statements about the **adequacy** of AMR surveillance systems in the **environmental health sector** in your country?

|  | Strongly agree (1) | Somewhat agree (3) | Somewhat disagree (4) | Strongly disagree (5) | I don't know (6) |
| --- | --- | --- | --- | --- | --- |
| The current surveillance systems for monitoring AMR are adequate (1) |  |  |  |  |  |
| The funding and resources allocated to AMR surveillance systems are adequate (2) |  |  |  |  |  |
| The AMR surveillance data is effectively utilised to inform policy and practice (3) |  |  |  |  |  |
| There is adequate training and support for personnel involved in AMR surveillance (4) |  |  |  |  |  |
| The AMR surveillance system is regularly updated to address new resistance patterns (7) |  |  |  |  |  |

End of Block: Surveillance systems

Start of Block: Block 10

26 Which of the following best describes the **collaboration** between the human, animal, and environmental sectors in **AMR surveillance** activities in your country?

- There is excellent collaboration among all three sectors. (1)
- There is good collaboration between the human and animal sectors, but not with the environmental sector. (2)
- There is some collaboration, but it is limited and not well-coordinated. (3)
- There is no significant collaboration among the sectors. (4)
- I don't know (5)

End of Block: Block 10

Start of Block: Antimicrobial usage Surveillance-human

27 Does your country have an **antimicrobial** **usage** **(AMU) surveillance** system in the **human health** sector?

- Yes (1)
- No (2)
- I don't know (3)

Display This Question:

If Does your country have an antimicrobial usage (AMU) surveillance system in the human health sector? = Yes

28 How **strongly** do you agree or disagree with the following statements about the **adequacy** of **AMU** surveillance systems in the **human health** sector in your country?

|  | Strongly agree (1) | Somewhat agree (2) | Somewhat disagree (3) | Strongly disagree (4) | I don't know (6) |
| --- | --- | --- | --- | --- | --- |
| The current surveillance systems for monitoring AMU are adequate (1) |  |  |  |  |  |
| The data collected by AMU surveillance systems is reliable and accurate (2) |  |  |  |  |  |
| The resources allocated to AMU surveillance systems are sufficient (3) |  |  |  |  |  |
| The AMU surveillance data are effectively utilised to inform policy and practice (4) |  |  |  |  |  |
| Record-keeping practices for antimicrobial use in the human health sector are reliable and consistent (5) |  |  |  |  |  |
| There is a well-established and interconnected electronic database system for AMU surveillance that operates effectively (6) |  |  |  |  |  |

| Page Break |  |
| --- | --- |

End of Block: Antimicrobial usage Surveillance-human

Start of Block: Antimicrobial usage Surveillance - Animal

29 Does your country have an **antimicrobial** **usage** **(AMU) surveillance** system in the **animal health** sector?

- Yes (1)
- No (2)
- I don't know (3)

Display This Question:

If Does your country have an antimicrobial usage (AMU) surveillance system in the animal health sector? = Yes

30 How **strongly** do you agree or disagree with the following statements about the **adequacy** of **AMU** surveillance systems in the **animal health** sector in your country?

|  | Strongly agree (1) | Somewhat agree (2) | Somewhat disagree (3) | Strongly disagree (4) | I don't know (6) |
| --- | --- | --- | --- | --- | --- |
| The current surveillance systems for monitoring AMU are adequate (1) |  |  |  |  |  |
| The data collected by AMU surveillance systems is reliable and accurate (2) |  |  |  |  |  |
| The resources allocated to AMU surveillance systems are sufficient (3) |  |  |  |  |  |
| The AMU surveillance data are effectively utilised to inform policy and practice (4) |  |  |  |  |  |
| Record-keeping practices for antimicrobial use in the animal health sector are reliable and consistent (5) |  |  |  |  |  |
| There is a well-established and interconnected electronic database system for AMU surveillance that operates effectively (6) |  |  |  |  |  |

End of Block: Antimicrobial usage Surveillance - Animal

Start of Block: Antimicrobial usage Surveillance - Plant

31 Does your country have an **antimicrobial** **usage** **(AMU) surveillance** system in the **environmental health** (e.g. Plant) sector?

- Yes (1)
- No (2)
- I don't know (3)

Display This Question:

If Does your country have an antimicrobial usage (AMU) surveillance system in the environmental heal... = Yes

32 How **strongly** do you agree or disagree with the following statements about the **adequacy** of **AMU** surveillance systems in the **environmental health** sector in your country?

|  | Strongly agree (1) | Somewhat agree (2) | Somewhat disagree (3) | Strongly disagree (4) | I don't know (6) |
| --- | --- | --- | --- | --- | --- |
| The current surveillance systems for monitoring AMU are adequate (1) |  |  |  |  |  |
| The data collected by AMU surveillance systems is reliable and accurate (2) |  |  |  |  |  |
| The resources allocated to AMU surveillance systems are sufficient (3) |  |  |  |  |  |
| The AMU surveillance data are effectively utilised to inform policy and practice (4) |  |  |  |  |  |
| Record-keeping practices for antimicrobial use in the plant health sector are reliable and consistent (5) |  |  |  |  |  |
| There is a well-established and interconnected electronic database system for AMU surveillance that operates effectively (6) |  |  |  |  |  |

| Page Break |  |
| --- | --- |

33 Which of the following best describes the **collaboration** between the human, animal, and environmental sectors in **AMU** **surveillance** activities in your country?

- There is excellent collaboration between all three sectors. (1)
- There is good collaboration between the human and animal sectors, but not with the environmental sector. (2)
- There is some collaboration, but it is limited and not well-coordinated. (3)
- There is no significant collaboration among the sectors. (4)
- I don't know (5)

End of Block: Antimicrobial usage Surveillance - Plant

Start of Block: Education and Awareness

34 Please indicate your **level of agreement** with the following statements about the **current status** in your country

|  | Strongly agree (1) | Somewhat agree (2) | Somewhat disagree (3) | Strongly disagree (4) | I don't know (5) |
| --- | --- | --- | --- | --- | --- |
| Doctors receive adequate training on antimicrobial stewardship (1) |  |  |  |  |  |
| Veterinarians receive adequate training on antimicrobial stewardship (11) |  |  |  |  |  |
| Policymakers are well informed about the importance of regulating antibiotic sales and use (6) |  |  |  |  |  |
| There are adequate public awareness campaigns addressing the misuse of antibiotics (2) |  |  |  |  |  |
| Farmers are aware of the need for responsible antibiotic use in animals. (3) |  |  |  |  |  |
| School children are taught about antimicrobial resistance and the importance of appropriate antibiotic use (4) |  |  |  |  |  |
| The media effectively disseminates information about AMR (7) |  |  |  |  |  |

End of Block: Education and Awareness

Start of Block: One Health

35 Are you aware of the One Health concept?

- Yes, I understand its principles well. (1)
- I have heard of it, but I'm not familiar with its details. (3)
- I'm aware of it but unsure of its relevance. (4)
- No, I have never heard of it before. (2)

36 If you are aware of this term, what does it mean to you?

________________________________________________________________

________________________________________________________________

________________________________________________________________

________________________________________________________________

________________________________________________________________

37 Have you undergone formal instruction or training pertaining to One Health throughout your professional journey?

- Yes (1)
- No (2)

38 Do you think introducing One Health principles into the education of professionals in your field would be advantageous?

- Yes (1)
- No (2)
- One Health principles are already taught in my field (4)

39 How useful do you think a One Health approach would be for tackling AMR issues in your country, whether or not you currently use this approach?

- Yes, very useful (1)
- Not very useful (2)
- Not useful at all (3)
- I am not aware of the One Health approach (4)

40 How confident are you in the collaboration between different stakeholders (e.g., healthcare professionals, veterinarians, policymakers) in addressing AMR in your country?

- Highly confident (3)
- Moderately confident (2)
- Slightly confident (4)
- Not confident at all (1)

| Page Break |  |
| --- | --- |

41 How important do you think the following stakeholders are in dealing with the issue of AMR?

|  | Highly important (4) | Moderately important (3) | Slightly important (2) | Not at all important (1) | I don't know (5) |
| --- | --- | --- | --- | --- | --- |
| Veterinarians (1) |  |  |  |  |  |
| Doctors (2) |  |  |  |  |  |
| Dentists (3) |  |  |  |  |  |
| Nurses (4) |  |  |  |  |  |
| Pharmacists (5) |  |  |  |  |  |
| Para-veterinary staff (veterinary nurses, animal handlers) (6) |  |  |  |  |  |
| Scientists and microbiologists (7) |  |  |  |  |  |
| Patients (8) |  |  |  |  |  |
| Livestock Farmers (9) |  |  |  |  |  |
| Animal breeders (10) |  |  |  |  |  |
| Pet owners (11) |  |  |  |  |  |
| Government and policymakers (12) |  |  |  |  |  |
| Professional associations (e.g. Medical/ Veterinary associations etc.) (13) |  |  |  |  |  |
| Global organisations (e.g. WHO, OIE) (14) |  |  |  |  |  |
| Pharmaceutical companies (15) |  |  |  |  |  |
| Media (16) |  |  |  |  |  |
| Politicians (17) |  |  |  |  |  |

End of Block: One Health

Start of Block: Block 9

42 If you think there are other stakeholders dealing with the issue of AMR that are not mentioned above, please write them below

________________________________________________________________

________________________________________________________________

________________________________________________________________

________________________________________________________________

________________________________________________________________

End of Block: Block 9
